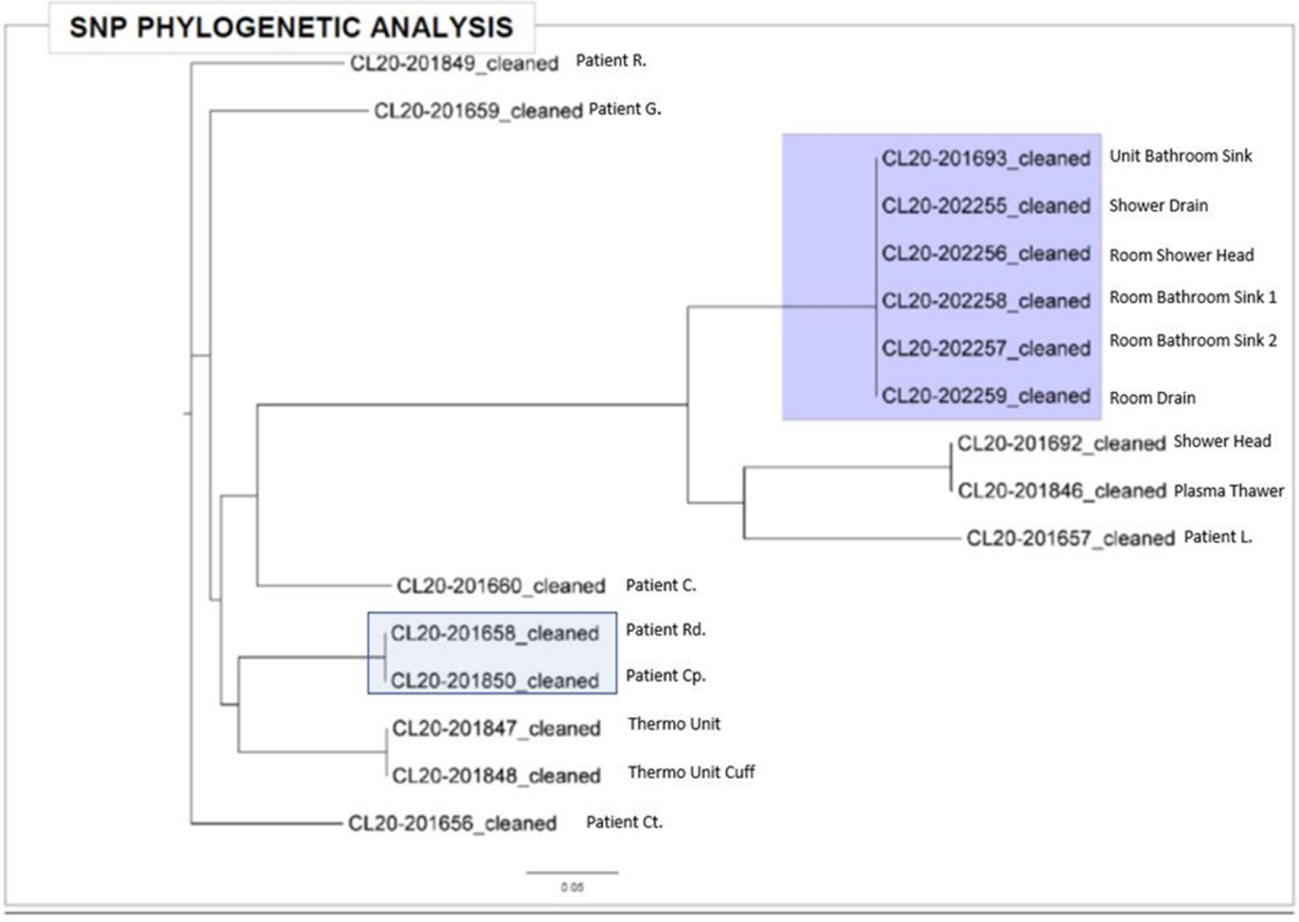# Outbreak of *Pseudomonas aeruginosa* Bacteremia Infections among Stem-Cell Transplant Patients Related to Change in Prophylaxis

**DOI:** 10.1017/ash.2021.46

**Published:** 2021-07-29

**Authors:** Kerrie VerLee, Chau Nguyen, Russell Lampen, Jim Codman, Tunisia Peters

## Abstract

**Background:** *Pseudomonas aeruginosa* outbreaks can originate from various sources and can cause severe complications in posttransplant patients. Antibiotic prophylaxis can decrease posttransplant infections; however, consideration must be given to *P. aeruginosa* coverage as we outline an outbreak among the stem-cell transplant (SCT) population. **Methods:** A multidisciplinary outbreak investigation was conducted to evaluate sources of contamination and changes in clinical processes. Positive blood cultures from SCT patients and environmental isolates were analyzed using whole-genome sequencing (WGS). Incidence density rates for *P. aeruginosa* blood cultures from January 2019 through October 2020 were calculated per 10,000 patient days and stratified by unit, specimen, and transplant type. Statistical analysis was calculated with significance at p < 0.05. **Results:** A cluster of 8 SCT patients was identified between May and September 2020. Moreover, 10 environmental samples were positive for *P. aeruginosa* including drains, water sources prior to the point-of-use (POU) filter and blood-bank thaw machines. Phylogenetic analysis revealed 1 cluster of 2 patients who shared the same room, 5 patients with unique *P. aeruginosa* isolates, and 2 separate clusters of environmental isolates with relatedness only to each other. Review of clinical processes showed a change from fluoroquinolone prophylaxis to cephalosporin in the spring of 2020. Also, 5 *P. aeruginosa* bacteremia infections occurred prior to June (11.78 cases per 10,000 patient days). During the period of cephalosporin use, 8 infections were identified (58.27 cases per 10,000 patient days) (*P =* .006). Following the restart of fluoroquinolone, zero infections have occurred to date, as of January 28, 2021. **Conclusions:** Discontinuation of fluoroquinolone prophylaxis was associated with *P. aeruginosa* bacteremia infections in SCT patients. Use of fluoroquinolone prophylaxis in SCT patients is protective from *P. aeruginosa* bacteremia infections. There have been no further infections in the following 3 months after the change back to the use of fluoroquinolone. Additionally, WGS showed that most patient isolates did not have a common source, suggesting that *P. aeruginosa* gastrointestinal colonization may play a role in seeding these bacteremia infections.

**Funding:** No

**Disclosures:** None

**Figure 1.**
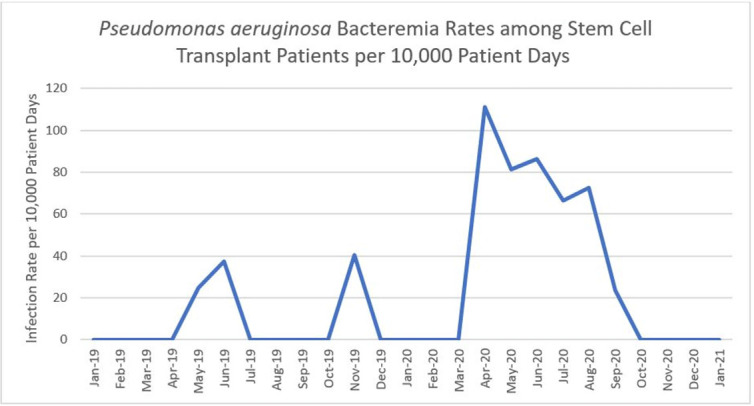

**Figure 2.**